# Pathogenicity and virulence of *Campylobacter jejuni*: What do we really know?

**DOI:** 10.1080/21505594.2024.2436060

**Published:** 2024-12-08

**Authors:** Zahra Omole, Nick Dorrell, Abdi Elmi, Fauzy Nasher, Ozan Gundogdu, Brendan W. Wren

**Affiliations:** Faculty of Infectious and Tropical Diseases, London School of Hygiene and Tropical Medicine, London, UK

**Keywords:** *Campylobacter jejuni*, virulence, bacterial gastroenteritis, fitness factors, surrogate infection models

## Abstract

*Campylobacter jejuni* is the leading cause of bacterial gastroenteritis and is a major public health concern worldwide. Despite its importance, our understanding of how *C. jejuni* causes diarrhoea and interacts with its hosts is limited due to the absence of appropriate infection models and established virulence factors found in other enteric pathogens. Additionally, despite its genetic diversity, non-pathogenic *C. jejuni* strains are unknown. Regardless of these limitations, significant progress has been made in understanding how *C. jejuni* uses a complex array of factors which aid the bacterium to survive and respond to host defences. This review provides an update on fitness and virulence determinants of this important pathogen and questions our knowledge on these determinants that are often based on inferred genomics knowledge and surrogate infection models.

## *Campylobacter jejuni* – an important pathogen but virulence is poorly understood

**Figure 1.**
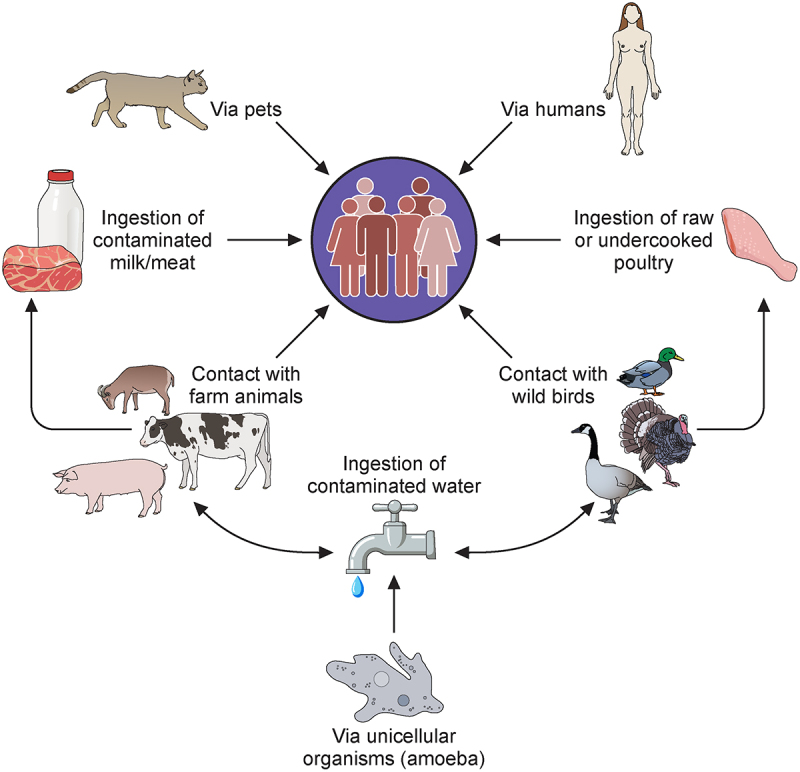
Diagrammatic representation of the known transmission routes of human infection by *Campylobacter jejuni*. arrows depict direction(s) of transmission.

*Campylobacter jejuni* is a microaerophilic bacterium, and despite its limited growth under normal atmospheric conditions, it is the leading cause of bacterial foodborne enteritis worldwide. *C. jejuni* colonises the gastrointestinal tract of various livestock with poultry being the major source of human infection [1]. Outbreaks of *C. jejuni* infections are also associated with the consumption of unpasteurised milk and untreated water but human-to-human transmission is rare [2–4] (Figure 1). Clinical symptoms of *C. jejuni* infection include watery or bloody diarrhoea accompanied by abdominal cramps, nausea and fever [5–7]. Although *C. jejuni* infection is self-limiting, occasionally post-infection sequelae can lead to neurological disorders including Guillain–Barré syndrome (GBS) [8–12]. According to the World Health Organisation, *C. jejuni* is responsible for 96 million cases of enteric infection globally each year and in low socio-economic environments *C. jejuni* infection remains a frequent cause of death in infants [13]. The economic burden is substantial, with an estimated annual cost of €2.4 billion in Europe [14] and up to $6.8 billion in the US [15].

Despite being the major causative agent of bacterial foodborne enteritis, as well as the availability of genomic information and genetic tools, the virulence factors necessary for *C. jejuni* to establish infection are not well characterised and some are still controversial. This is largely due to inconvenient or inappropriate infection models and also because *C. jejuni* lacks the classical virulence factors, including toxins and the classical Type III Secretory Systems (T3SSs), found in other enteric bacterial pathogens. A further gap in our knowledge is the lack of an explanation for the high prevalence of this major pathogen which does not grow well under atmospheric conditions. This has been referred to as the *Campylobacter* conundrum [16]. The high prevalence of *C. jejuni* was attributed to various factors such as large size of *C. jejuni* population in animal reservoirs, its ability to persist in a non-culturable but viable state [16] and its potential interactions with amoebae [17]. These factors may contribute significantly to its persistence and widespread occurrence in various environments and hosts, particularly livestock. Although *C. jejuni* thrives in the gut of birds and the consumption and handling of poultry is a major source of human infection, this cannot explain the high rates of human infection. Despite these limitations, we provide an overview of the *C. jejuni* lifecycle and virulence factors in the context of pathogenicity with reference to genomic data and surrogate infection models. For the purposes of this review, we define a virulence factor as a protein or macromolecular structure that contributes to the ability of the pathogen to cause disease and a fitness factor as a protein or macromolecular structure that, whilst not required for virulence, offers a competitive advantage to the pathogen during infection.

## *Campylobacter jejuni* – a sugar rich pathogen

**Figure 2.**
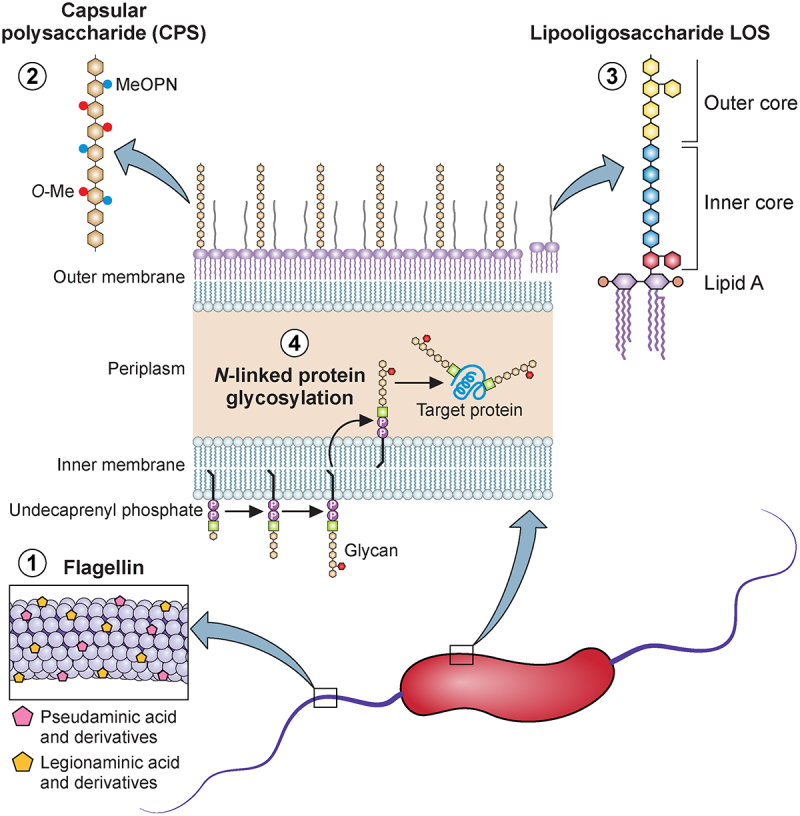
Schematic structures of *Campylobacter jejuni* sugar components implicated in virulence.

*C. jejuni* was amongst one of the first organisms to be fully sequenced which provided the itinerary of potential determinants to investigate (approx. 1600 gene products) [18]. An immediate feature of the sequence of *C. jejuni* strain NCTC11168 was the abundance of glycan structures including two independent glycosylation systems, the lipo-oligosaccharide (LOS) and a previously undiscovered capsular polysaccharide (CPS) (see Figure 2). These four glycan structures are present in all strains and are each likely to play a significant role in the survival and pathogenesis of *C. jejuni*. Notably, the surface exposed flagellin glycan, LOS and CPS are all phase variable and genetically diverse among *C. jejuni* strains. By contrast the periplasmic *N*-linked glycosylation system is absolutely conserved.

1. The flagellar proteins are modified through *O*-linked glycosylation with pseudaminic acid and its derivatives, as well as legionaminic acid and its derivatives, which are essential for flagellar assembly and bacterial motility [19,20]. 2. The capsular polysaccharide (CPS) forms a protective layer around the bacterium, with modifications including 2-*O*-methylphosphoramidate (MeOPN) and *O*-methyl (*O*-Me) groups, enhancing its structural diversity and protective functions [20]. 3. The lipooligosaccharide (LOS) is a variable glycolipid component of the outer membrane, crucial for host immune modulation and bacterial pathogenicity [21–23]. 4. Additionally, *C. jejuni* exhibits *N*-linked glycosylation, where a conserved heptasaccharide core is attached to asparagine residues in proteins, playing a significant role in protein stability and bacterial adaptability within the host [24,25]. Figure was generated in Biorender.

The *O*-linked glycosylation system modifies the flagellin (see below) and was known to be important for motility prior to genome sequencing. However, the discovery of a *N*-linked general glycosylation system, which post-translationally modifies over 100 *C. jejuni* proteins was unexpected and was the first described *N*-linked glycosylation system found in a bacterium [26]. The precise role of the *N*-linked glycosylation system in *C. jejuni* and in other non-pathogenic *Campylobacter* species is still unclear, 25 y after its discovery. However, *N*-linked glycosylation deficient mutants are less able to adhere to cultured cells and colonise chickens, but such mutants are pleiotropic and the direct role remains to be determined [27].

The LOS is located on the surface of *C. jejuni* cells and has been shown to be important in the evasion of the host immune system, adhesion to and invasion of epithelial cells and survival in hostile environments. It has been proposed that molecular mimicry between the LOS structure of some *C. jejuni* strains and human neuronal gangliosides can produce cross-reactive antibodies responsible for the autoimmune complication such as GBS [28,29]. The LOS structure is highly variable with many strains having sialic acid modifications [21]. It is proposed that the LOS sialylation contributes to the invasiveness of epithelial cell lines as a mutation in the *C. jejuni* LOS sialyltransferase *cst-II* gene triggers a decrease of invasion ability compared with the wildtype strain [30]. It is thought that the sialic acid present in LOS of *C. jejuni* leads to an increase in the activation of human dendritic cells through Toll-like receptor 4 signalling and subsequent B cell proliferation [31]. Disease severity has been suggested to be related to the presence of sialic acid in the LOS composition [22,23]. However, Ellstrom *et al*. showed that only 23% of the isolates that caused bacteraemia in humans carry LOS containing sialic acid modifications and found no association between the presence of sialic acid and the severity of the symptoms of campylobacteriosis [32].

The *C. jejuni* CPS is found on the outermost layer of the cell surface and is composed of a sugar with an unusual configuration [24,25,33]. *C. jejuni* CPS is required to resist complement-mediated killing [34,35], invade human intestinal epithelial cells (IECs) *in vitro* [36,37], colonisation of chicks [38] and diarrhoeal disease in ferrets [34]. The O-methyl phosphoramidate (MeOPN) modification has also been demonstrated to be essential for complement resistance. Additionally, it has been demonstrated the *C. jejuni* capsule prevents excessive cytokine production by dendritic cells [39].

## *C. jejuni* flagella: function and virulence

*C. jejuni* has flagella at each pole of the cell and each flagellum is a multifunctional organelle. Flagella facilitate the bacterium in penetrating the viscous mucosa lining of the human IECs and in reaching the distal ileum, jejunum and colon. Through chemotaxis the flagella enable *C. jejuni* to avoid noxious agents and move towards nutrient sources [40]. Thus, *C. jejuni* flagella promote motility, chemotaxis and host colonisation. In addition, *C. jejuni* flagella also promote adhesion to and invasion of human IECs *in vitro* [41–44], biofilm formation [45] and non-flagellar protein export [46–48]. The latter enables *C. jejuni* to secrete ~57 predicted proteins, of which ~six are putative virulence-associated, including those termed *Campylobacter* Invasion Antigens (Cia) [46–48]. To date four Cia proteins have been identified. The role of CiaB is still unclear; however, *ciaB* mutants exhibit reduced invasion of IECs and do not export effector proteins, suggesting a role in the secretion of Cia proteins [48]. CiaC plays a role in bacterial invasion as *ciaC* mutants exhibit reduced invasion of IECs and CiaC has been shown to be delivered into IECs [48]. CiaD binds directly to the host cell protein IQGAP1, resulting in displacement of RacGAP1 from the IQGAP1 complex, increasing the activity of Rac1 which plays a role in *C. jejuni* invasion [49,50]. CiaI plays a role in *C. jejuni* survival within IECs with *ciaI* mutants exhibiting reduced intracellular replication but wild-type levels of adhesion and invasion [51]. Expression of Cia proteins occur upon contact of *C. jejuni* with IECs [52] and the human bile salt sodium deoxycholate (SD) has been shown to up-regulate the expression of all four *cia* genes [53]. Cia proteins co-opt components of the focal complex and the MEK/ERK signalling pathway to trigger membrane ruffling to promote *C. jejuni* invasion of IECs [48]. Secretion of Cia proteins and other virulence factors is thought to be dependent on a functional flagellar [46,48,54].

*C. jejuni* flagellar synthesis and glycan modification involves over 50 flagellum-related genes. The flagellar filament is composed of subunits of FlaA and FlaB proteins. The flagellar subunit FlaA rather than FlaB is essential for *C. jejuni* motility because mutation of the *flaA* gene results in the generation of non-flagellated and non-motile cells [55,56]. By contrast, the mutation of *flaB* has no impact on *C. jejuni* flagella synthesis and motility. The role *of C. jejuni* flagella in chick colonisation is further confirmed through mutation of the genes encoding the flagellar motor proteins MotA and MotB which are essential for the rotation of the flagella. A *C. jejuni motAB* mutant produced non-motile cells with a full-length flagellum that is unable to rotate, thus unable to colonise chicks [57].

Like other *Campylobacter* species, *C. jejuni* flagellin is conserved and undergoes post-translational modification by *O*-linked glycosylation which adds conserved pseudaminic acid, and in some strains, additional legionaminic acid and their derivatives [19,20]. The major flagellin FlaA is glycosylated at ~19 serine and threonine sites, with mutations to specific sites resulting in autoagglutination defects and impaired motility due to flagella destabilisation [58,59]. Flagellin glycosylation is considered a microbe associated molecular pattern (MAMP) which bind to pathogen recognition receptors (PRRs) on various phagocytes, including those found in warm-blooded mammals [60,61]. A distinctive feature of the *C. jejuni* flagellar filament is the ability to escape immune interaction with Toll-like receptor 5 (TLR5), found on the basolateral side of the human IECs. This immune evasion was attributed to mutations in *C. jejuni* flagellar TLR5 epitope resulting in significantly weakened binding to TLR5 [62,63]. Additionally, the *C. jejuni* flagellin glycosylation locus includes a homopolymeric-tract-containing gene, *Cj1295*, which may contribute to host evasion or avoidance of phage predation by spontaneous antigenic variation [64].

## *C. jejuni* putative adhesins and invasion

Breaching of the human IEC barrier leading to internalisation of *C. jejuni* is considered to play an important role in *C. jejuni* pathogenesis [65]. *C. jejuni* has been shown to cross the epithelial barrier, invading underlying tissues such as the lamina propria and enter the bloodstream, via both transcellular and paracellular transmigration routes [66]. The mechanism of *C. jejuni* invasion is thought to demonstrate features of both the “trigger” and “zipper” cell entry mechanisms [67]. *C. jejuni* has been shown to induce membrane ruffling, typical of the “trigger” mechanism, with speculation that secretion of Cia proteins through the flagella in a T3SS-like manner could support the utilisation of the “trigger” mechanism for cell entry by *C. jejuni* [67,68]. *C. jejuni* also utilises adhesin–receptor interactions to enable invasion via endocytosis, a characteristic of the “zipper” mechanism [67,69]. These adhesin–receptor interactions are thought to in part be mediated by CadF (Campylobacter adhesion to fibronectin) and FlpA (Fibronectin-like protein A), which are known to interact with fibronectin (Fn) found on the basolateral surface of human IECs [69–71]. Fn is a large extracellular matrix glycoprotein, expressed on the plasma membrane of human IECs. Fn binding triggers biochemical and mechanical signalling via integrin binding, thus acts as a scaffolding platform that modulate *C. jejuni* invasion [72]. CadF has a molecular mass of 37 kDa and comprises 326 amino acids [73]. Four amino acids at position 134–137 exposed on the surface of CadF have been shown to be responsible for the binding to Fn [74]. FlpA has a molecular mass of 46 kDa harbouring surface exposed Fn type III domains [75,76] and is reported to be composed of three Fn III-like repeats D1, D2 and D3, binding Fn via a motif within the D2 repeat [77]. CadF and FlpA have been extensively characterised to play a role in *C. jejuni*–host cell interactions. Mutation of *cadF* was shown to reduce the ability of *C. jejuni* to colonise the caecum of newly hatched Leg-horn chickens, as well as reducing bacterial adherence and invasion of human INT 407 cells *in vitro* [78]. CadF was up-regulated in the outer membrane of oxygen-acclimatised *C. jejuni* and a *cadF* mutant showed reduced adhesion to inert surfaces compared to the wild-type strain [79]. Reduced binding to INT 407 human IECs by both *cadF* and *flpA* mutants has also been observed [71]. In addition, mutation of *flpA* reduced the ability of *C. jejuni* to adhere to chicken LMH hepatocellular carcinoma epithelial cells *in vitro*, adhere to INT 407 cells and colonise broiler chickens compared with a *C. jejuni* wild-type strain [75].

In addition to CadF and FlpA, the other well-studied *C. jejuni* adhesin is the Jejuni lipoprotein A (JlpA) [80]. JlpA has a molecular mass of 42 kDa and comprises 372 amino acids. Mutation of *jlpA* results in reduced adhesion to Hep-2 cells [80]. JlpA interacts directly with surface heat shock protein (HSP) 90a, which is constitutively expressed in epithelial cells under normal conditions but can be up-regulated under stress conditions [81]. Binding of JlpA to HSP90a results in the activation of several different signalling pathways, including NF-kB and p38 MAP kinase [81].

*C. jejuni* major outer membrane protein (MOMP) is a pore-forming protein that has been shown to bind to both fibronectin and INT 407 cell membranes [82]. However, as MOMP has also been shown to play a role in the transport of ions across the bacterial cell wall [83], due to the essentiality of this function, it has not been possible to confirm the role in adhesion through construction of a defined mutant [84]. Additional *C. jejuni* surface-exposed adhesins, such as *Campylobacter* adhesion protein A (CapA), PEB1 [85,86] and PEB4 [87] have all been investigated. These adhesins which also play a role in *C. jejuni* adherence to human and chicken IECs are a good example of the multifactorial ability of *C. jejuni* virulence mechanisms. It is hypothesised that these different *C. jejuni* adhesins are required in the multiple steps of infection. First, to adhere to the mucosal layer at the luminal side of IECs and then to adhere to the basolateral side of the IECs following either transcellular or paracellular transmigration [66]. Additionally, the diversity of adhesins in *C. jejuni* strains highlights the adaptability of the bacterium and must contribute to the ability to colonise different hosts and environments [88].

## *C. jejuni* outer membrane vesicles

Secretion of virulence factors is the major mechanism by which enteric bacterial pathogens interact with host cells to enhance the survival of the bacteria and/or damage the host cell. Secretion mechanisms utilised by enteric bacterial pathogens often require intimate contact with the host cell, such as the T3SS, the Type IV secretion system (T4SS) and the Type VI secretion system (T6SS) that deliver bacterial proteins and non-proteinous moieties (such as fragments of peptidoglycan) directly into the host cytoplasm. Surprisingly *C. jejuni* lacks many of the virulence-associated secretion systems possessed by other enteric bacterial pathogens. No dedicated T3SS has been identified in *C. jejuni*; however, the flagellar apparatus can export non-flagellar proteins in a T3SS-like manner [18] and whilst both T4SSs and T6SSs have been identified in some *C. jejuni* strains, strains lacking both T4SS and T6SS still cause disease in humans [48]. Studies have suggested that the secretion of Cia proteins and other virulence factors is dependent on a functional flagellar [46,48,54]. However, there is as yet no evidence that the secretion of virulence factors via the *C. jejuni* flagellar can facilitate the direct delivery of bacterial proteins into host cells as is the case with the classical T3SSs in other enteric bacterial pathogens.

Rather than secrete virulence factors into the surrounding milieu where they could be degraded by host proteases, many Gram-negative bacterial pathogens utilise outer membrane vesicles (OMVs) as a mechanism of delivering active proteins into host cells [89]. In this way, virulence factors are not secreted individually, but as multiple factors delivered in a concerted manner. OMVs play roles in establishing colonisation of the host, delivering virulence factors into host cells and modulating the host responses for many Gram-negative bacterial pathogens [89]. Toxin delivery mediated by OMVs is now recognised as a potent virulence mechanism [89].

Both non-pathogenic and pathogenic Gram-negative bacteria constitutively release OMVs [90]. Bacterial OMVs are discrete, closed outer membrane blebs produced by growing cells and not a product of cell lysis or cell death. OMVs are small, spherical, proteoliposomes with an average diameter ranging from 20–500 nm [90]. Many virulence factors that are periplasmic proteins are enriched in OMVs, including enterotoxigenic *E. coli* (ETEC) heat labile enterotoxin (LT) [91]. Recently, studies have reported a diverse range of bacterial pathogens that release OMVs into their extracellular space, where OMVs have been associated to play an important role in pathogenesis [92]. For example, OMVs secreted by *Pseudomonas aeruginosa* have been shown to deliver multiple virulence factors (including beta-lactamase, alkaline phosphatase, haemolytic phospholipase C and the Cif toxin) directly into the cytoplasm of airway epithelial cells [93].

The role of *C. jejuni* OMVs during host-pathogen interactions has been studied over the past 15 y. The first study to report cytotoxic activity associated with *C. jejuni* OMVs was published in 2009, where biologically active cytolethal distending toxin (CDT) was shown to be secreted within OMVs produced by the *C. jejuni* 81–176 wild-type strain [94]. All three CDT subunits (the CdtA, CdtB, and CdtC proteins) were detected by immunogold labelling and electron microscopy of OMVs [94]. The DNase activity of the CdtB subunit induces eukaryotic cell cycle arrest at the G2/M stage with resultant cessation of cell division [95]. Isolated OMVs were shown to exert the cell distending effects typical of CDT on the human intestinal cell line HCT8 [94]. *C. jejuni* CDT exerts multiple effects including the release of interleukin 8 (IL-8) from IECs [96,97]; however, OMVs isolated from a 11,168 h *cdtA* mutant induced IL-8 to the same extent as wild-type OMVs, indicating OMV-mediated induction of IL-8 is independent of CDT [98]. A recent study has now shown that the three CDT subunits are internal to OMVs and do not appear to play a role in OMV-host cell interactions [99]. Glycan array studies demonstrated that OMVs bind complex host cell glycans and share receptor binding specificities with live *C. jejuni* bacteria for fucosyl GM1 ganglioside, P1 blood group antigen, sialyl and sulphated Lewis^x^ [99]. OMVs produced by the *C. jejuni* 11168 h wild-type strain possess cytotoxic activity against both Caco-2 IECs and *Galleria mellonella* larvae, which are often used as a convenient, if limited, model of *C. jejuni* infection [98]. *C. jejuni* OMVs also induced a host immune response from T84 IECs, which was not affected by pre-treatment of OMVs with either proteinase K or polymyxin B prior to co-incubation with IECs [98]. Pre-treatment of IECs with methyl-β-cyclodextrin (MβCD) partially blocked the OMV-induced host responses, indicating a role for lipid rafts in the host cell plasma membrane during interactions with *C. jejuni* OMVs [98]. A recent study has investigated the mechanisms of host cell-specific OMV uptake in more detail, showing that intracellular perfusion of *C. jejuni* OMVs is mediated by cytosolic as well as multiple endocytic uptake processes [100]. Furthermore, target cell-specific preferential uptake of *C. jejuni* OMVs was demonstrated using human and avian cells as two different host targets [100].

The first proteomic analysis of *C. jejuni* 11168 h OMVs identified 151 specific proteins including periplasmic and outer membrane-associated proteins, but also many determinants known to be important in survival and pathogenesis including CDT [98]. Subsequent proteomic analyses to characterise the protein content of *C. jejuni* OMVs have focused on different strains (81–176 and NCTC11168) and/or different conditions (growth at 42°C compared to 37°C, growth in the presence of physiological concentrations of bile salts, exposure to oxygen or polymyxin B stress), indicating that *C. jejuni* OMV cargo is modulated in response to different environments [101–104]. A diverse range of *C. jejuni* proteins from different bacterial compartments have been identified associated with OMVs, including many flagellar-associated proteins, adhesins including CadF, FlpA and JlpA, *N*-linked glycoproteins and efflux pump sub-units CmeA and CmeC [98,101–104].

Human bile salts have been shown to induce virulence gene expression in enteric bacterial pathogens. SD enhances *C. jejuni* virulence gene expression [53] whilst sodium taurocholate (ST) induces *Vibrio cholerae* virulence gene expression [105]. Physiological concentrations of ST do not have an inhibitory effect on *C. jejuni* growth until the early stationary phase [106]. Co-culture of *C. jejuni* with 0.1% or 0.2% (w/v) ST stimulates OMV production, increasing both lipid and protein concentrations [106]. ST‐OMVs exhibit a different protein profile compared to OMVs isolated in the absence of ST [106]. ST‐OMVs exhibit enhanced cytotoxicity and immunogenicity to T84 IECs as well as enhanced killing of *G. mellonella* larvae [106]. ST increases the level of mRNA transcripts of the *cdtABC* operon that encodes CDT [106]. *C. jejuni* regulates OMV production in response to host gut signals through changes in expression of the maintenance of outer membrane lipid asymmetry (MLA) pathway and ST down-regulates the MLA pathway resulting in increased OMV production [107].

## Serine proteases secreted in *C. jejuni* OMVs

Proteomic analysis also identified three serine proteases HtrA, Cj0511 and Cj1365c associated with 11,168 h OMVs [98,104]. HtrA is a bifunctional protease that is highly conserved in *Campylobacter* species [65]. HtrA has two PDZ domains (Post-synaptic density protein 95, Drosophila disc large tumour suppressor and Zonula occludens-1 protein domain) in addition to a trypsin-like domain. An important study highlighted the crucial role of HtrA in *C. jejuni* temperature tolerance and survival at high temperatures of up to 44°C [108]. Mutation of *htrA* reduced the ability of *C. jejuni* to withstand increased levels of oxygen and different external stresses [109]. These early studies on HtrA provided the basis for an in-depth investigation of the diverse functions within the physiological and stress response pathways of *C. jejuni*. HtrA regulates *C. jejuni* protein folding and co-ordinates the subsequent removal of misfolded proteins, as well as the refolding of aggregated proteins, thereby emerging as a pivotal component in the regulation of *C. jejuni* protein quality [110]. The dual role of HtrA, functioning as both a protease and a chaperone, emphasises this crucial function as a molecular co-ordinator [111]. However, functional significance of HtrA extends beyond protein quality control. Mutations in the chaperone domain reduce capacity of *C. jejuni* to adhere to and invade human IECs [112]. Moreover, mutation of *htrA* reduces the cytotoxicity of *C. jejuni* in *G. mellonella* larvae [113]. These findings underscore the important bifunctionality of HtrA in shaping pathophysiology and virulence of *C. jejuni*. *C. jejuni* secretes HtrA in its oligomeric form into the surrounding media, resulting in cleavage of E-cadherin, occludin, and claudin-8 in IECs [106,114–118]. The functional properties linked to the cleavage properties of HtrA are attributed to the serine motif in S197A [119].

The other two serine proteases associated with 11,168 h OMVs are both known to have a role in the pathophysiology of *C. jejuni* [120]. Cj0511 is a serine protease that has been identified as a *N-*linked glycoprotein. Cj0511 is crucial for the establishment of *C. jejuni* in chickens [121]. In addition to this, Cj0511 plays a pivotal role in the release of α-dextran when *C. jejuni* cells are exposed to pancreatic amylase [122]. The release of α-dextran in turn facilitates biofilm formation, enhances interactions with human epithelial cells and augments the pathogenicity of *C. jejuni* in the *G. mellonella* infection model [122]. Furthermore, the enhanced release of α-dextran enhances the *C. jejuni* colonisation within the ileum of chickens [122]. The Cj1365c protease is classified as a serine protease possessing a domain resembling that of a subtilisin family [106]. The catalytic triad of this substance consists of histidine, aspartate and serine [120]. The N-terminal of Cj1365c contains a S8 peptidase domain and the C-terminal functions as an autotransporter domain, enabling the translocation of the protease across cellular membranes.

C. *jejuni* OMVs were shown to possess significant proteolytic activity that was reduced by pre-treatment with serine protease inhibitors [98]. OMVs isolated from either *htrA*, *Cj0511* or *Cj1365c* mutants exhibited significantly reduced protease activity, which could be further reduced by the addition of serine protease inhibitors [98]. *C. jejuni* OMVs can cleave both E-cadherin and occludin; however, this cleavage is reduced with OMVs pre-treated with serine protease inhibitors and with OMVs isolated from *htrA* or *Cj1365c* mutants [117]. ST increases the level of mRNA transcripts of *htrA*, *Cj0511* and *Cj1365c* and ST‐OMVs possess increased proteolytic activity [106]. Co-culture with ST significantly enhances the OMVs induced cleavage of E‐cadherin and occludin [106]. Co-incubation of T84 monolayers with 11,168 h OMVs was shown to result in a visible reduction in both E-cadherin and occludin [117]. *C. jejuni* OMVs also cleave the major endoplasmic reticulum chaperone protein BiP/GRP78 and this activity was shown to be associated with only the Cj1365c protease [106]. Co-incubation of T84 IECs with 11,168 h OMVs during co-culture with live *C. jejuni* (MOI 100:1) resulted in increased numbers of interacting bacteria at over 24 h [117]. The effect on bacterial invasion was even more pronounced with an increase of almost 50% in the number of intracellular bacteria over 24 h [117]. A recent study has investigated the effects of soluble recombinant HtrA and HtrA associated with OMVs on polarised Caco-2 and MDCK cells, compared to live *C. jejuni* cells actively expressing HtrA [123]. In contrast to earlier studies, only live *C. jejuni* were shown to cause damage to both tight and adherens junction components of polarised Caco-2 and MDCK cells, such as occludin, claudin-8, and E-cadherin [123]. It is noteworthy that both the OMV isolation methodology and the cell lines used differ from those used in earlier studies, so further work will be required to clarify these conflicting observations.

*C. jejuni* OMVs have emerged as a crucial vehicle that can secrete potent virulence factors. This in turn enhances *C. jejuni* virulence adaptability, such as increasing bacterial adhesion to and invasion of host cells, as well as contributing to bacterial survival against host defences.

## *Campylobacter jejuni* – stress response

During its life cycle, *C. jejuni* transitions many environments. To adapt to various exogenous and internal stresses, *C. jejuni* relies on a sophisticated array of regulatory proteins, including OmpR‐type response regulators such as *Campylobacter* oxidative stress regulator (CosR) [124], peroxide-sensing regulator (PerR) [125] and Multiple Antibiotic Resistance Regulator (MarR‐type regulators designated regulator of response to peroxide, RrpA and RrpB) [126,127].

CosR, a versatile regulator, orchestrates the expression of genes crucial for *C. jejuni’s* fitness and survival, particularly those involved in macromolecule biosynthesis, metabolism, gene regulation and oxidative stress response [124,128]. CosR controls the expression of stress response-related proteins such as LuxS, rubredoxin oxidoreductase/rubrerythrin, alkyl hydroperoxide reductase (AhpC) and superoxide dismutase (SodB). SodB is the most important enzyme in the detoxification of O^2−^ and contributes to *C. jejuni* chicken colonisation and intracellular survival [129,130]. Similarly, PerR is an independent regulator and governs peroxide stress genes along with *sodB* [130,131].

During human infection, *C. jejuni* undergoes a confined proliferation and shifts its physiology to adapt to different stress conditions while at the same time rewiring its metabolism [132,133]. One of the first stresses that *C. jejuni* endures in the human host is body temperature, which is known to influence virulence gene expression [101,134,135]. Some of *C. jejuni* oxidative stress proteins also function as a response against temperature stress [136–138] and there are heat shock proteins [138,139]. Also, In the human and avian hosts, *C. jejuni* experiences stresses such as low pH stresses during passage through the gastro-intestinal (GI) tract [140,141] and the innate immune system-mediated metal ion toxicity by the mechanism of RNA thermometer (RNAT) regulation [142], highlighting the importance of temperature sensing in this pathogen’s survival strategies.

*C. jejuni* lacks the glycolytic enzyme phosphofructokinase of the Embden–Meyerhof–Parnas pathway [143–145] and it utilises a limited number of amino acids as carbon and energy sources [146]. This unusual capacity to metabolise a few glucogenic amino acids enables *C. jejuni* to efficiently incorporate nutrients into anabolic processes for the synthesis of lipids, proteins and nucleic acids [147]. It is suggested *C. jejuni* pathogenesis involves the ability of the bacterium to rewire its metabolic adaptations as much it also relies on specific virulence factors targeting host proteins [147–149].

A prevailing belief has been that *C. jejuni* possess proteins to sense the human host metabolites, such as bile acids, free fatty acids, amino acids and vitamins. *C. jejuni* is also equipped with a mechanism to sense metabolites derived from gut microbiota, such as bacteriocins, short-chain fatty acids and quorum-sensing autoinducers. Recently, a study has elucidated during *in vivo* growth that *C. jejuni* recognises spatially different intestinal regions by sensing lactate [150] and short-chain fatty acids such as acetate and butyrate and then suitably modifies transcription of colonization factors [151]. The *C. jejuni* BumSR two component regulatory system is an indirect sensor of butyrate BumS which functions as a phosphatase, via a noncanonical mechanism for signal transduction rather than as a kinase, to control the activity of the cognate BumR response regulator [152]. Additionally, we have also demonstrated that host metabolites such as bile salt sodium taurocholate increases the demand on *C. jejuni* proteases and CDT toxin function [106].

## Virulence factors present in *C. jejuni* subpopulations and type VI secretion system (T6SS)

The sequencing of multiple genomes of *C. jejuni* isolates have demonstrated strain-specific genetic diversity, particularly in its secretion systems. While the presence of a functional type IV secretion system (T4SS) remains controversial [48,153], multiple studies have shed light on the prevalence and significance of the type VI secretion system (T6SS) within specific *C. jejuni* lineages [154–156]. The T6SS, essential for niche adaptation and virulence, has been identified in *C. jejuni* strains associated with both animal hosts and human infections [157,158].

Structurally, the T6SS constitute an envelope-spanning complex facilitating the secretion of effector molecules [159,160]. Among these, TssD emerges as a critical component, serving dual roles as both a structural protein and an effector [64,161–163]. Its deletion results in the loss of T6SS activity, impacting crucial aspects such as host colonisation and cell adherence [164,165]. Notably, T6SS-positive strains have been linked to severe clinical outcomes, including bloody diarrhoea and increased pathogenicity [163,166,167].

However, the role of T6SS and effectors in *C. jejuni* pathogenesis is understudied. While some studies report haemolysis and cytotoxic activities associated with T6SS, these phenotypes are likely linked to unique or strain-specific effectors indicating the need for further investigation [163,164,168–170]. Moreover, the T6SS facilitates bacterial competition within ecological niches [171,172], providing a competitive growth advantage for *C. jejuni* in complex microbial communities.

Beyond its role in virulence, the T6SS also plays a crucial role in stress response modulation, aiding in the bacterium’s survival against environmental challenges such as oxidative stress and bile salt exposure [162,164]. Environmental factors, such as bile salt concentration, can influence the efficacy of T6SS-mediated responses, highlighting its adaptive nature [173].

The T6SS emerges as a key determinant in *C. jejuni* pathogenesis and strain transmissibility, presenting avenues for future research into therapeutic interventions. Understanding the intricate interplay between *C. jejuni* and its secretion systems provides valuable insights into combating this pervasive foodborne pathogen.

### Surrogate infection models

Various models have been used to study *C. jejuni*, including human volunteers [41], non-human primates [174], mice [175], ferrets [153] and chickens [176,177]. Human volunteers and non-human primates are impractical in most circumstances, whilst the mouse model does not result in diarrhoea and is unsatisfactory when compared to infection with other enteric bacterial pathogens such as Salmonella. *G. mellonella* infection models have been used as an alternative to mammalian or avian models due to reduced costs and no ethical requirement for it use [178]. However, the insect larvae are an unnatural host for *C. jejuni* which limits conclusions that can be drawn regarding true host-pathogen interactions. Both *in vitro* avian and mammalian cell lines have been widely used to study *C. jejuni* interactions with host cells; however, the aerobic conditions that such experiments are usually performed under do not accurately mimic the conditions under which these interactions take place. When *C. jejuni* interactions with Caco-2 cells were investigated using a Vertical Diffusion Chamber model, a 9-fold increase in interacting and 80-fold increase in intracellular *C. jejuni* were observed after 24 h co-culture under microaerobic conditions at the apical surface, compared to results under aerobic conditions [179].

To address the *Campylobacter* conundrum, we and others have investigated amoebae, such as *Acanthamoebae* interactions with *C. jejuni* [180–183]. Emerging evidence strongly suggests that *Acanthamoebae* may transiently harbour *C. jejuni* and shield the bacteria from the external environment [183]. Despite being considered a thermophilic organism, *C. jejuni* readily grows at 30°C, an optimal temperature for *Acanthamoebae*, and can maintain metabolic activity at lower temperatures (Hazeleger et al., 1998b). This characteristic, and the ubiquitous presence of *Acanthamoebae*, implies a shared ecological niche. Recent discoveries highlight significant similarities in properties between *Acanthamoebae* and *C. jejuni* animal hosts, demonstrating comparable survival mechanisms when infecting both *A. castellanii* and warm-blooded hosts [184]. *Acanthamoebae* appear to pre-adapt *C. jejuni*, enhancing subsequent invasion of human epithelial cell lines and re-invasion of *Acanthamoebae* species [183]. In contrast, interactions with the soil-dwelling *Dictyostelium discoideum* result in the rapid digestion of *C. jejuni* shortly after internalisation [185]. This prompts the question of whether a co-evolutionary connection exists between *C. jejuni* and *Acanthamoebae*. Consequently, investigating the interactions between host and prey provides a valuable model for gaining insights into *C. jejuni* adaptation and infection, as well as exploring ecological and evolutionary dynamics.

## Conclusions and future perspective

Despite enormous efforts over many decades, we still have little understanding of how *C. jejuni* causes diarrhoea in humans? Why are humans and primates so sensitive to *C. jejuni* infection with as few as a hundred cells causing diarrhoea [41]? By contrast, avians can tolerate trillions of *C. jejuni* cells without signs of overt disease. From an evolutionary perspective, *C. jejuni* is from the epsilon proteobacteria subdivision of bacteria that invariably have *N*-linked glycosylation pathways [186]. Epsilon proteobacteria are frequently found in deep-sea vents and survive in diverse environments including co-existing with amoebae and thriving in the gut of avians. Humans are accidental *C. jejuni* hosts that happen to be exquisitely sensitive to harbouring the accidental passenger.

Future studies should include the refinement, optimisation and normalisation of both experimental design and protocols that represent ideal settings to study the interaction of defined *C. jejuni* strains with defined host cells, allowing researchers to reproduce data. Unsurprisingly, there are a plethora of *C. jejuni* studies that use experimental approaches that give an insight into the selected role of *C. jejuni* putative virulence factors in stress survival, adhesion, invasion and intracellular survival, yet few provide information about the function of such putative virulence factors.

Recent developments in the understanding of *C. jejuni* pathogenesis have combined several experimental approaches that link the functional characterisation of potential virulence factors. The use of surrogate infection models, such as the amoeba host infection model, the mouse enteropathy and diarrhoeal model [187], *in vitro* avian and mammalian cell lines, hold the potential to significantly enhance our understanding of *C. jejuni* pathogenesis and virulence factors. *In vitro* cell line models provide controlled environments for studying the interactions between *C. jejuni* and host cells, shedding light on the intricacies of its survival and adaptation mechanisms. The integration of virulence characterisations with spatial analysis at various time points and considering the genetic variability among strains is essential to advance our understanding of *C. jejuni* pathogenesis. It is crucial that all the above are integrated with interdisciplinary approaches such as omics-based approaches to achieve our aims and objectives. This comprehensive approach will allow researchers to not only investigate the molecular aspects of virulence but also understand how these factors manifest over time and vary between strains. By bridging the gap between virulence assessments and spatial-temporal analyses, we can gain deeper insights into the dynamic nature of *C. jejuni* infections, ultimately contributing to more effective strategies for prevention and intervention.

## Data Availability

Data sharing is not applicable to this article as no new data were created or analysed in this study.
